# Comparison of Protocols to Test Peptide Stability in Blood Plasma and Cell Culture Supernatants

**DOI:** 10.1021/acsptsci.4c00503

**Published:** 2024-10-14

**Authors:** Anna Kohler, Eva-Maria Jülke, Jan Stichel, Annette G. Beck-Sickinger

**Affiliations:** Institute of Biochemistry, Faculty of Life Sciences, Leipzig University, 04103 Leipzig, Germany

## Abstract

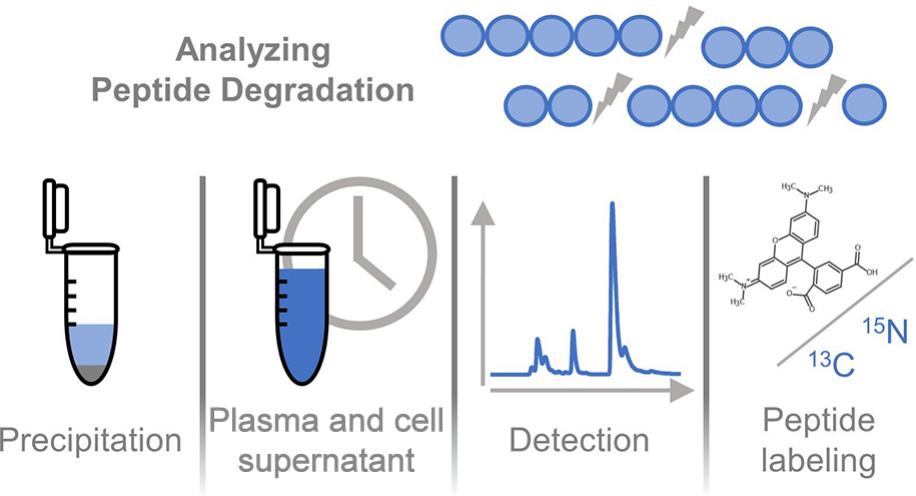

Due to their high specificity, peptides are promising candidates in drug development, but fast degradation often limits their biological activity. Thus, a short half-life is one of the major challenges in the development of new peptide therapeutics. Moreover, the enzymatic cleavage of peptides can be a reason for misleading results in biological assays. Peptide stability assays typically consist of incubation, precipitation, and detection steps. However, the current methods differ greatly regarding these three steps, thus limiting the compatibility. Here, we systematically evaluate different parameters of peptide stability assays. First, we quantified and compared the analyte loss during the precipitation of plasma proteins. Especially, broadly used precipitation by strong acids was found to be unsuitable, while mixtures of organic solvents preserved more peptides for further analysis. Next, the stability of four fluorescently labeled model peptides was analyzed in blood plasma and two different cell culture supernatants. Strong variation in the degradation dynamics and patterns was found. Finally, we evaluated the role of fluorescent labeling on peptide stability and compared results to peptides with isotopic labels, underlining the individual advantages of both methods. Altogether, the data provide important parameters for analyzing and comparing the peptide stability.

Peptides are promising tools for the development of innovative therapeutics that revolutionize the treatment of various diseases. They offer remarkable cell and target specificity compared to those of small molecules, making them ideal candidates for targeted therapies with reduced side effects. Furthermore, peptide therapeutics often have lower production complexity compared to protein-based therapeutics representing the golden mean between less specific small molecules and biopharmaceuticals.^1^ However, the success of peptide therapeutics depends on the stability within the complex and dynamic environment of the human body, especially on rapid proteolytic cleavage or renal clearance. Thus, naturally occurring peptides provide only a starting point for the design of stable peptide therapeutics.

Different strategies have been developed to overcome in vivo degradation of peptides, such as introduction of noncanonical amino acids, lipidation, or cyclization. One prominent example is the stabilization of glucagon-like peptide 1 (GLP-1) by the attachment of fatty acids.^2,3^ For the development of Semaglutide, different linker and fatty acid combinations were evaluated to ensure good receptor activity and strong serum albumin binding.^4^ The attachment of octadecadienoic acid (Odd) as used for Semaglutide was adapted for other peptides like peptide YY_3–36_ (PYY_3–36_)^5^ and adrenomedullin (ADM) to increase half-life.^6^ Stabilization through peptide cyclization based on disulfide bridges can be found in setmelanotide or vasopressin.^7,8^

Furthermore, peptide stability can vary significantly between different species due to variations in the composition of proteases and peptidases. For example, the valine-citrulline linker, commonly used in shuttling systems based on selective cleavage by endosomal cathepsin B, is stable in human blood plasma, but fast extracellular cleavage was observed by carboxylesterase 1c in murine blood.^9^ Interestingly, introduction of glutamic acid prior to the valine-citrulline linker enhances stability of the linker in mouse plasma.^10^

Although testing peptide stability is an essential part of peptide therapeutics development, there are many inconsistent protocols in the literature.^11−16^ An underestimated problem is peptide loss during sample preparation, which leads to a low concentration and detectability of degradation products. Furthermore, low peptide stability under assay conditions can be a source of misleading results. Thus, we compared various peptide precipitation methods by using four different representative 6-carboxytetramethylrhodamine (Tam)-labeled peptides and quantified peptide loss during sample preparation. We also examined peptide stability in human blood plasma and two different cell culture supernatants with reversed-phase high-performance liquid chromatography (RP-HPLC) and matrix-assisted laser desorption/ionization time-of-flight mass spectrometry (MALDI-ToF-MS). Additionally, we tested the degradation of isotope-labeled peptides by liquid chromatography–electrospray ionization–mass spectrometry (LC/MS) in comparison to the fluorescence-based approach. Significant differences were found to depend on the applied protocols, which are crucial for the future approach to the development of stable peptide therapeutics.

## Experimental Section

### Comparison of Different Precipitation Conditions

Human blood plasma was provided by “Institut für Transfusionsmedizin”, Medical Center, Leipzig University and was evaluated by the ethics commission of the Medical Faculty of Leipzig University (“Entwicklung stabilisierter Peptidanaloga für die Wirkstoffforschung”, Aktenzeichen 527/21-ek).

Using low-bind tubes, 10 mM peptide solutions in dimethyl sulfoxide (DMSO, Sigma-Aldrich) were diluted in human blood plasma/Dulbecco’s phosphate-buffered saline (DPBS, Biowest; 1:1, v/v) to a 10 μM final concentration and directly precipitated in “precipitation **A**” 2× volume acetonitrile (ACN, VWR)/ethanol (EtOH, PanReac AppliChem, 1:1, v/v), “precipitation **B**” 2× volume ACN, “precipitation **C**” 1× volume acetonitrile overnight at −20 °C, or “precipitation **D**” 1% trichloroacetic acid (TCA, Sigma-Aldrich, v/v) for 20 min at room temperature. The peptide samples and peptide stock reference were dissolved to a concentration of 1.1 μM peptide in 20% ACN + 0.1% formic acid (FA, Sigma-Aldrich) in H_2_O (v/v/v) and filtered through Costar Spin-X tubes (0.22 μm, Sigma-Aldrich). For LC-MS (LC/MSD-IQ System, Agilent Technologies), 50 μL of the filtrate was injected on an AdvanceBio Peptide Plus column (50 × 2.1 mm, 100 Å, 2.7 μm, flow rate 0.3 mL/min, Agilent Technologies; gradient: 1 min isocratic, 5–60% ACN + 0.1% FA in H_2_O + 0.1% FA over 18 min), and the maximal total ion count (TIC) was determined relative to the reference (statistical analysis: 2-way ANOVA using the Tukey posthoc test with GraphPad Prism, Version 10.1.2).

### Investigation of Peptide Stability in Human Blood Plasma

Peptide solutions were diluted as described above and incubated at 37 °C. Samples were precipitated in 2× ACN/EtOH (1:1, v/v) at −20 °C overnight and filtered through Costar Spin-X tubes. The solutions of Tam-labeled peptides diluted with H_2_O (1:1, v/v) were analyzed by RP-HPLC using a linear gradient of eluent B (0.1% TFA in H_2_O) in eluent A (0.08% TFA in ACN) over 40 min at 40 °C on a VariTide RPC column (250 mm × 4.6 mm, 200 Å, 6 μm, flow rate 1 mL/min, Agilent Technologies). The relative amount of intact peptide was determined by fluorescence intensity (extinction 525 nm, emission 572 nm). Half-life was calculated by one-phase decay in Prism 10. Furthermore, eluting peptides were collected, lyophilized, and analyzed by MALDI-ToF-MS. Solutions with isotope-labeled peptides were lyophilized, reconstituted in H_2_O/20% ACN/0.1% FA (v/v/v), and centrifuged for 5 min at 13,000 × *g*. The supernatant was used for nano-LC/MS/MS analysis with a nLC1000 UHPLC system (Thermo Fisher Scientific) coupled to the EASY-Spray ion source of an Orbitrap Elite mass spectrometer (Thermo Fisher Scientific). Details of the procedure can be found in the supplementary section (Method S2).

### Investigation of Peptide Stability in the Cell Culture Supernatant

HEK-293 cells were cultivated under standard conditions in Dulbecco’s modified Eagle’s medium (DMEM, Biowest)/Ham’s F12 (Biowest, 1:1, v/v) with 15% fetal bovine serum (FBS, Sigma-Aldrich). HEK-293 cells were detached with trypsin/ethylenediaminetetraacetic acid (Lonza) and seeded in a poly-d-lysine hydrobromide (Merck, 0.1 mg/mL)-coated 96-well cell culture microplate (Greiner Bio-One, 100,000 cells/well). The cells were grown under standard conditions. Calu-3 cells were cultured in minimum essential medium (MEM, Biowest) with 10% FBS (v/v), 2 mM glutamine, 1 mM sodium-pyruvate (Lonza), and 1× nonessential amino acids solution (Lonza). For detachment, Calu-3 cells were washed twice with DPBS and incubated with TrypLE (Gibco) for 15–20 min at 37 °C. For differentiation, 200,000 cells/cm^2^ were seeded in 24-well inserts (Greiner Thincert, translucent, 0.4 μm pore size) and cultured for 2 days under standard conditions. Cell differentiation was supported by culturing cells on an air–liquid interface for 10–14 days and monitored by measurement of transepithelial electrical resistance. For the stability assay, peptides (10 mM DMSO stock) were diluted in phenol red-free DMEM with 15% FBS for HEK-293 cells or phenol red-free DMEM/Ham’s F12 + 0.1% casein (Fluka) for Calu-3 cells to a 10 μM final concentration. The medium was replaced with the peptide solutions. Cells were incubated under standard conditions, and after respective time points, samples were precipitated in 2× EtOH/ACN (1:1, v/v). Further sample preparation and analysis were performed as described above.

## Results and Discussion

To evaluate the effects of different protocols used to measure peptide stability, we tested peptides with varying lengths from 9 to 36 amino acids. Differing peptide hydrophobicity was achieved by incorporating various fatty acids such as lauric acid or octanoic acid, and Tam or isotope labels were introduced in the peptide sequence (Table 1). All peptides were synthesized by solid-phase peptide synthesis (SPPS) by the Fmoc/*tert*-butyl strategy with purities ≥95%. Details on peptide synthesis and analytics can be found in the Supporting Information (Method S1 and Table S1).

**Table 1:** Sequences of Synthesized Peptides^a^

	peptide	sequence
**1**	[K^4^(Tam),F^7^,P^34^]-pNPY	YPSK(Tam)PDFPGEDAPAEDLARYYSALRHYINLITRPRY- NH_2_
**2**	[K^27^(Lau),K^31^(Tam)]-sNPY_(27–36)_	K(Lau)INPK(Tam)-Bip-RLRY-NH_2_
**3**	[Dpr^3^,K^16^(Tam)]-Ghr	GS-Dpr(Oct)-FLSPEHQRVQQRK(Tam)ESKKPPAKLQPR
**4**	[Dpr^3^,K^16^(Tam),K^20^(Odd)]-Ghr	GS-Dpr(Oct)-FLSPEHQRVQQRK(Tam)ESKK(Odd)PPAKLQPR
**5**	[F^7^,K^18^(Tam),P^34^]-pNPY	YPSKPDFPGEDAPAEDLK(Tam)RYYSALRHYINLITRPRY- NH_2_
**6**	[F^7^,P^34^]-pNPY	YPSKPDFPGEDAPAEDLARYYSALRHYINLITRPRY- NH_2_
**7**	[F^7^,G^9^(^13^C_2_,^15^N),A^18^(^13^C_3_),L^30^(^13^C_6_,^15^N),P^34^]-pNPY	YPSKPDFPG(^13^C_2_,^15^N)EDAPAEDLA(^13^C_3_)RYYSALRHYINL(^13^C_6_,^15^N)ITRPRY- NH_2_

a Bip = biphenylalanine; Dpr = 2,3-diaminopropionic acid; Lau = lauric acid; Tam = 6-carboxytetramethylrhodamine; Oct = octanoic acid; Odd = octadecanoic diacid. Peptides 1 and 3 were described previously.^17,18^

### Different Precipitation Conditions for Sample Preparation

**Figure 1:**
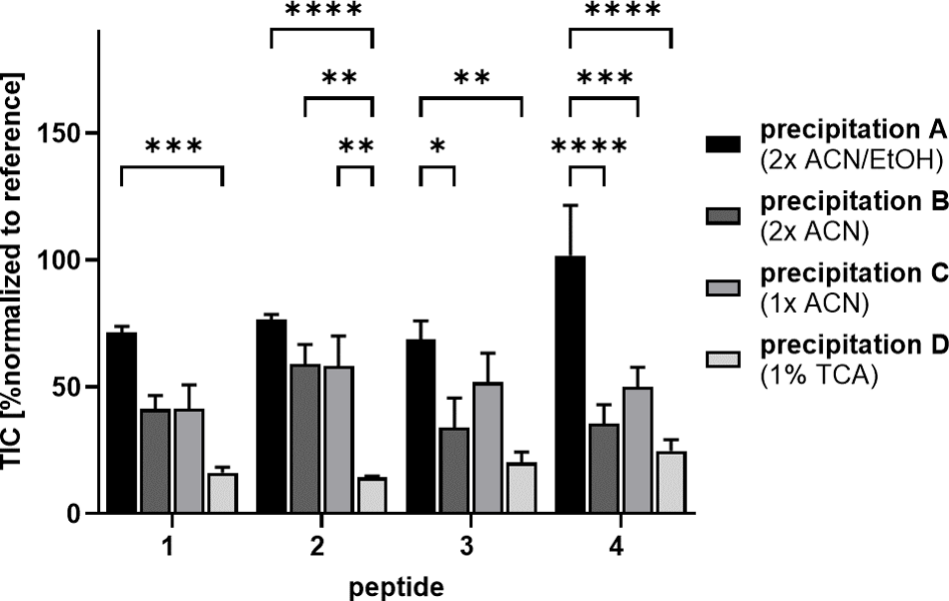
Effects of precipitation methods on the detected amount of peptide. A 10 μM peptide solution in human blood plasma/DPBS (1:1) was mixed with different amounts of ACN, EtOH, or TCA to precipitate plasma proteins prior to LC-MS analysis. The amount of peptide was determined by the TIC relative to a peptide reference sample dissolved in 20% ACN in H_2_O. **1:** K^4^(Tam),F^7^,P^34^]-pNPY; **2:** [K^27^(Lau),K^31^(Tam)]-sNPY_(27–36)_; **3:** [Dpr^3^,K^16^(Tam)]-Ghr; **4:** [Dpr^3^,K^16^(Tam),K^20^(Odd)]-Ghr; *n* = 4. Significance: *: *p* < 0.05; **: *p* ≤ 0.01; ***: *p* ≤ 0.005; ****: *p* ≤ 0.001.

Protein precipitation from blood plasma or other biological fluids used for stability assays is a critical step to prevent further degradation and reduce background signals. Therefore, organic solvents or strong acids are added to induce precipitation. To analyze the influence of the used precipitation method on the analyte, we evaluated four different precipitation methods (precipitation **A**–**D**) inspired from the literature and tested them with four example peptides varying in length and structural characteristics. A Tam-labeled NPY analogue [K^4^(Tam),F^7^,P^34^]-pNPY (peptide **1**), the NPY-derived decapeptide [K^27^(Lau),K^31^(Tam)]-sNPY_(27–36)_ (peptide **2,** Lau = lauric acid), a ghrelin analogue [Dpr^3^,K^16^(Tam)]-Ghr (peptide **3**), and a stabilized ghrelin analogue [Dpr^3^,K^16^(Tam),K^20^(Odd)]-Ghr (peptide **4**) were incubated in human blood plasma. After the precipitation of plasma proteins, the amount of peptide remaining in the supernatant was quantified by the maximal TIC relative to a reference sample (Figure 1). For all tested peptides, most of the compound was lost by precipitation of **D** with TIC reduction by at least 75% compared to the reference. Relative signal intensities after precipitation of **C** decreased to around 50%. If at all, using precipitation **B** only slightly decreased signals for ghrelin-based peptides **3** and **4** but not for NPY-based derivatives **1** and **2**. At least for peptide **2**, ACN-based precipitations **B** and **C** performed significantly better than TCA-based precipitation **D**. Best signal intensities were obtained by precipitation **A** for all peptides. However, for peptides **1**–**3**, signal intensities were still reduced by around 25% compared to the reference, indicating that the peptides partly precipitated with all evaluated methods. This coprecipitation of the peptides resulting in decreased signal intensities in the subsequent MS analysis is barely avoidable and should be reduced as much as possible by using an appropriate precipitation method. Even though published studies often use TCA in concentrations up to 10%,^11,12^ our data indicate that this method is not optimal for the isolation of peptides from biological fluids. Additionally, the evaluated precipitation methods showed different effects on the tested peptides, suggesting that precipitation also depends on individual peptide properties. Overall, precipitation **A** was the most suitable procedure for the tested peptides to eliminate plasma proteins from the samples with minimized peptide loss. Therefore, this method was used for further experiments.

### Comparison of Blood Plasma from Different Donors

**Figure 2:**
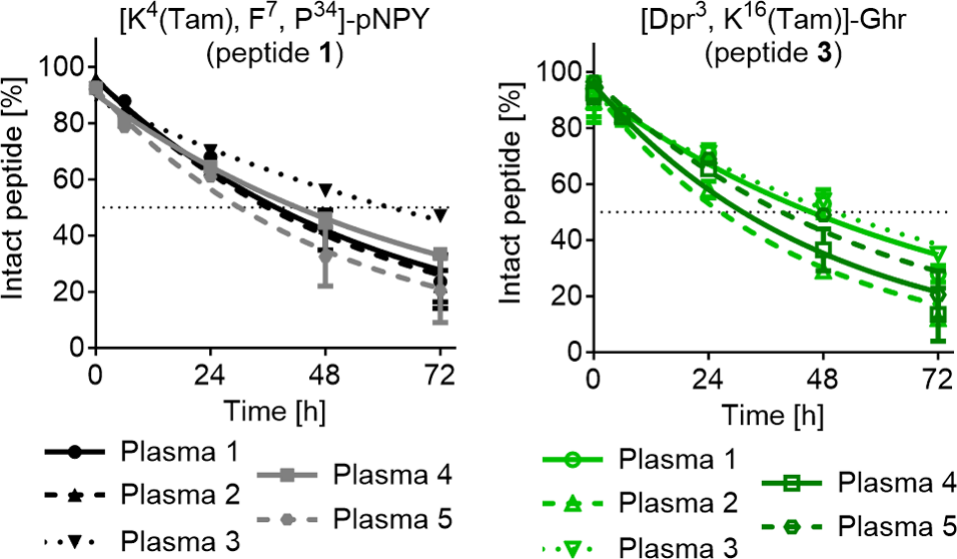
Comparison of peptide stability in blood plasma from different donors. Stability of Tam-labeled peptides was assessed in human blood plasma/DPBS (1:1). Peptide solutions (10 μM) were incubated at 37 °C and 500 rpm. The amount of intact peptide was measured by the area under the curve in RP-HPLC detecting fluorescence. Values represent the mean ± SEM of *n* ≥ 2 independent experiments.

As not only the precipitation method but also the used plasma batch may influence the measured peptide stability, we tested human blood plasma from five different donors to quantify the degradation of peptides **1** and **3** (Figure 2). Interestingly, three plasma samples showed similar half-lives for peptide **1**, ranging from 34.0 h (95% confidence interval (CI) = 25.6–46.6 h) to 40.3 h (95% CI = 32.0–52.2 h). In contrast, the half-life determined in plasma 4 for the same peptide was slightly increased to 49.4 h (95% CI = 45.5–53.8 h) and clearly extended in plasma 3 (*t*_1/2_ = 74.6 h; 95% CI = 64.7–87.4 h). Plasma 3 also showed the slowest degradation dynamics for peptide **3** (*t*_1/2_ = 57.4 h 95% CI = 49.5–67.7 h). The shortest half-life of peptide **3** was found in plasma 2. Taken together, the different half-lives of both peptides show a difference of up to 2-fold between the plasma batches. Furthermore, those variations are inconsistent between the two tested peptides. Generally, peptide stability assays are performed under conditions with the peptide concentration not serving as the limiting factor, but the degradation rate correlating linearly with plasma, and consequently enzyme concentration.^19^ Typically, enzyme concentrations vary between biological samples, as also indicated by the varying half-lives obtained for the same peptide in different blood plasma. This problem is further underlined by previous studies determining a half-life comparable to our data for fluorescently labeled [F^7^,P^34^]-pNPY analogues, but in undiluted human blood plasma.^17,20^ This suggests a more rapid degradation compared to our studies but might be explained by plasma batch variations. We suggest testing peptide stability in different plasma samples to gain insight into the biological variability of peptide degradation dynamics. A better understanding of the rate of peptide degradation in different individuals is particularly important for the development of effective peptide therapeutics.

### Stability of Tam-Labeled Peptides in Blood Plasma

**Figure 3:**
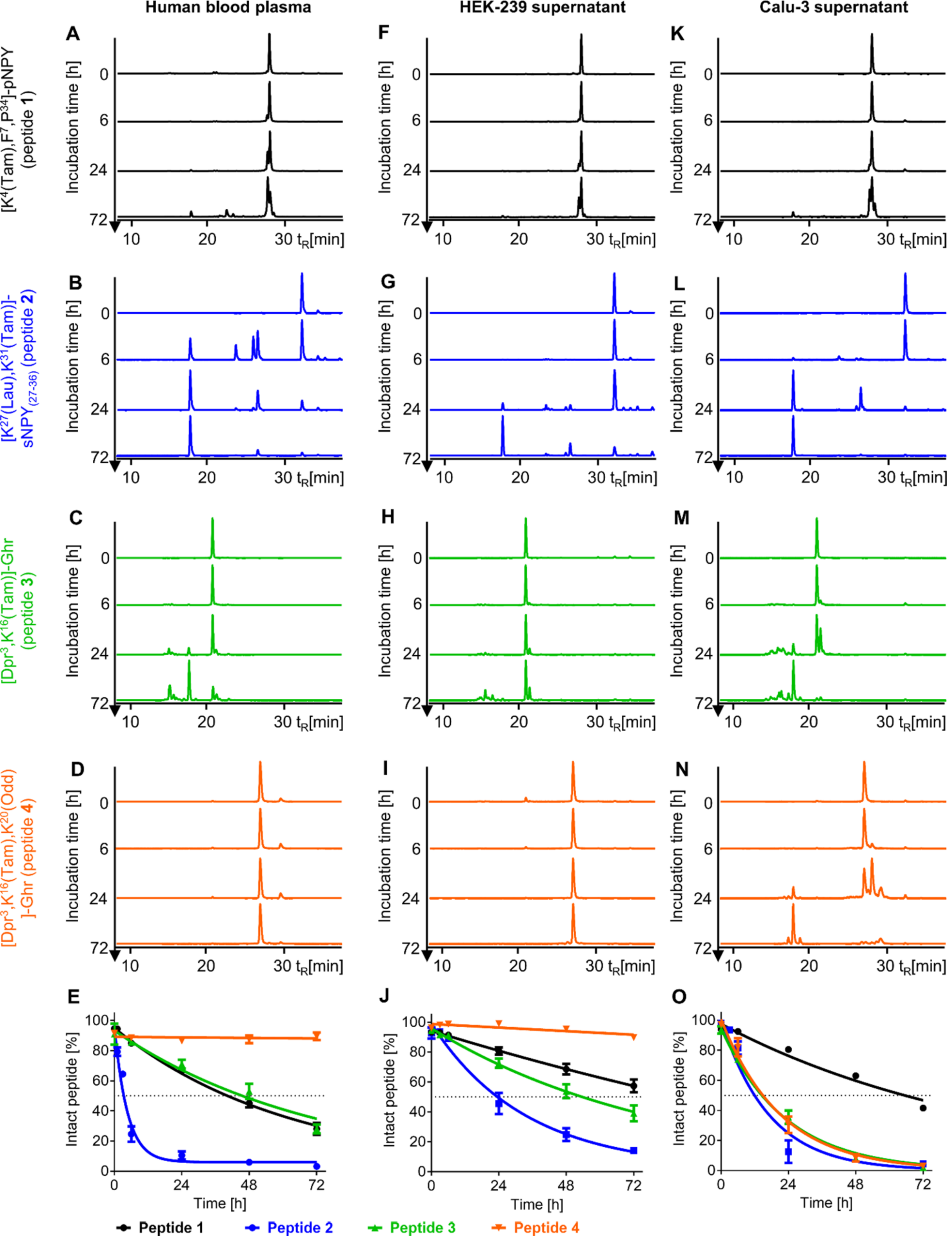
Stability of Tam-labeled peptides was accessed in human blood plasma/DPBS (1:1, A–E), HEK-293 (F–J), and Calu-3 supernatant (K–O). Degradation of [K^4^(Tam),F^7^,P^34^]-pNPY (black), [K^27^(Lau),K^31^(Tam)]-sNPY_(27–36)_ (blue), [Dpr^3^,K^16^(Tam)]-Ghr (green), and [Dpr^3^,K^16^(Tam),K^20^(Odd)]-Ghr (orange) was analyzed by RP-HPLC with fluorescence detection (data shown as representative of *n* ≥ 2 independent experiments), and the amount of intact peptide was quantified by area under the curve (mean ± SEM of *n* ≥ 2 independent experiments; E, J, O).

Peptide drugs are often applied by subcutaneous or intravenous injection and reach their targets by distribution along the cardiovascular system. Therefore, stability in the bloodstream is a key factor for peptide therapeutics, and blood plasma is used to investigate the degradation of peptides. Here, we incubated the Tam-labeled peptides **1**–**4** in human blood plasma (Figure 3A–D) and determined degradation dynamics (Figure 3E). Peptide **1** had a blood plasma half-life of 43.5 h (95% CI = 39.2–48.5 h). In contrast, peptide **2** was rapidly metabolized (*t*_1/2_ = 3.2 h, 95% CI = 2.6–4.1 h). Many cleavage products were detected already after 6 h in blood plasma (Figure 3B), and after 72 h, only 3% of peptide **2** was intact. For peptide **3**, the plasma half-life was calculated to be 50.5 h (95% CI 39.8–66.7 h). In contrast, barely any degradation was observed for peptide **4** with around 90% intact peptide after 72 h (Figure 3D). Peptide stability in blood plasma is a first indicator for in vivo stability and is crucial for the development of peptide therapeutics, as peptide lead structures often lack metabolic stability and show fast renal clearance.^1,21^ Peptidases in the bloodstream can increase degradation, while peptide binding components can increase their stability. Most prominently, serum albumin can reversibly bind peptides by hydrophobic moieties, like fatty acids.^22,23^

Lauric acid, a C12 acid, is attached to Lys^27^ of the short NPY analogue **2** to increase G-protein activation in comparison to short NPY without attachment, but the stability of peptide **2** was never tested before.^24−26^ Previous studies have shown that this C12 acid can bind up to seven sites in human serum albumin, suggesting that it might stabilize covalently bound peptides.^27^ However, peptide **2** showed the lowest plasma stability of the tested compounds. Previous studies with GLP-1 analogues demonstrated that attachment of C12 fatty acids has reduced serum albumin affinities compared to longer fatty acids.^4,28^ In contrast to lauric acid, attachment of Odd extended the plasma half-life of various peptides like GLP-1, PYY, and ADM analogues.^4−6^ Here, this principle was adapted to peptide **4**. The increased stability of peptide **4** in blood plasma compared to compound **3** is caused by the strong binding of Odd to serum albumin.

### Stability of Tam-Labeled Peptides in the Cell Supernatant

With varying peptidase expression in different tissues and biological fluids, peptide stability can also change. To evaluate such changes, we have further tested peptides **1**–**4** in the HEK-293 (Figure 3F–J) and Calu-3 supernatants (Figure 3K–O). For peptides **1**–**3**, the stability in the HEK-293 supernatant was higher than that in blood plasma (Figure 3J). The strongest stability difference was observed for peptide **2** with a half-life of 23.3 h (95% CI = 14.8–44.3 h) in the HEK-293 supernatant compared to 3.2 h in blood plasma. Only peptide **4**, which was barely degraded in blood plasma, showed similar stability in the HEK-293 supernatant with degradation of less than 10% over 72 h. Peptide **4** was more stable compared to peptide **3** without Odd with a half-life of 57.1 h (95% CI = 48.9–68.2 h) in the HEK-293 supernatant. In contrast, the half-life of peptide **3** was shorter in the Calu-3 supernatant with 15.8 h (95% CI = 13.8–18.0 h) than in the other two tested fluids. Furthermore, peptide **4** half-life of 14.8 h (95% CI = 12.0–18.1 h) was in the same range as peptide **3**. The stability of peptides **1** and **2** was only slightly lower in the Calu-3 supernatant than in the HEK-293 supernatant. Besides half-lives, we also compared the cleavage products detected by MALDI-ToF MS from the HPLC eluents (Figure S1). Peptide **1** showed similar cleavage patterns across the three biological fluids with some *N*-terminally or *C*-terminally slightly truncated degradation products and only a few strong *C*-terminal truncation. Peptide **2** exhibited similar cleavage patterns in all tested fluids and strong *N*-terminal and *C*-terminal truncation up to the Tam-labeled K^31^. Like in peptide **2**, in peptide **3**, *N*- and *C*-terminal degradation sites were detected in similar amounts. For the stabilized ghrelin analogue **4**, no cleavage was observed in blood plasma or the HEK-293 supernatant, while strong *C*- and *N*-terminal truncation was observed in the Calu-3 supernatant.

Stabilization caused by the binding of fatty acid moieties to albumin has been observed in the HEK-293 supernatant, where the medium contained serum albumin from FBS. In contrast, the medium was not substituted with FBS for the differentiated Calu-3 cells. Thus, the stabilizing effect of the Odd serum albumin interaction is missing, and peptide **4** is degraded as fast as peptide **3**.

While binding to plasma proteins is a more indirect stabilization method, specific degradation sites within the sequence can be stabilized by backbone modifications or disturbance of the peptidase recognition site, as by the replacement of Ser^3^ by Dpr in ghrelin.^29^ In the Calu-3 supernatant, the highly similar peptides **3** and **4** had different degradation patterns. This might be explained by different precipitation of the fragments by the additional lipidation in peptide **4**, as discussed above. However, the same main cleavage sites were found in both peptides. *N*-terminal truncation of peptide **1** is known to be mainly caused by dipeptidyl-peptidase 4 (DPP-4), which cleaves *N*-terminal Xaa-Pro dipeptides.^30,31^ Also, cleavage after Arg^35^ in [F^7^,P^34^]-pNPY has already been reported previously.^20^ Cleavage after Pro^34^ in peptide **2** was observed in all assays with differing ratios indicating varying peptidase occurrence in the tested fluids and may be caused by cathepsin X and peptidyl-dipeptidase A or B activity.^31^ The high occurrence of this cleavage position in the Calu-3 supernatant in contrast to the other biological fluids indicates the high activity of the responsible peptidase in this cell line. This highlights the relevance of individual stability checks, depending on the context of the scientific assay setup. Stability trends can vary between different conditions and might promote misleading interpretations of cell-based assays. As the peptide solutions were prepared with fresh media, the secreted peptidases first had to be enriched in the cell supernatant, resulting in prolonged half-lives. While degradation is less important for short-time cellular signaling assays, e.g., calcium-flux assay, which are measured within seconds, it gains importance with extended incubation times as used for toxicity assays or shuttling systems.^32^ Besides stability assays, the individual cell lines might be used to mimic degradation from individual tissues. Calu-3 cells as a model system of the respiratory epithelium and known to show mucus segregation^33^ can give insights into early degradation of peptides upon nasal application. In contrast, subcutaneous injection leads to deposition of peptides in fat tissue, so the degradation in adipocytes such as murine 3T3-L1 or human SGBS cells could be of interest.

### Stabilizing Effect of Tam Label Depends on Positioning

**Figure 4:**
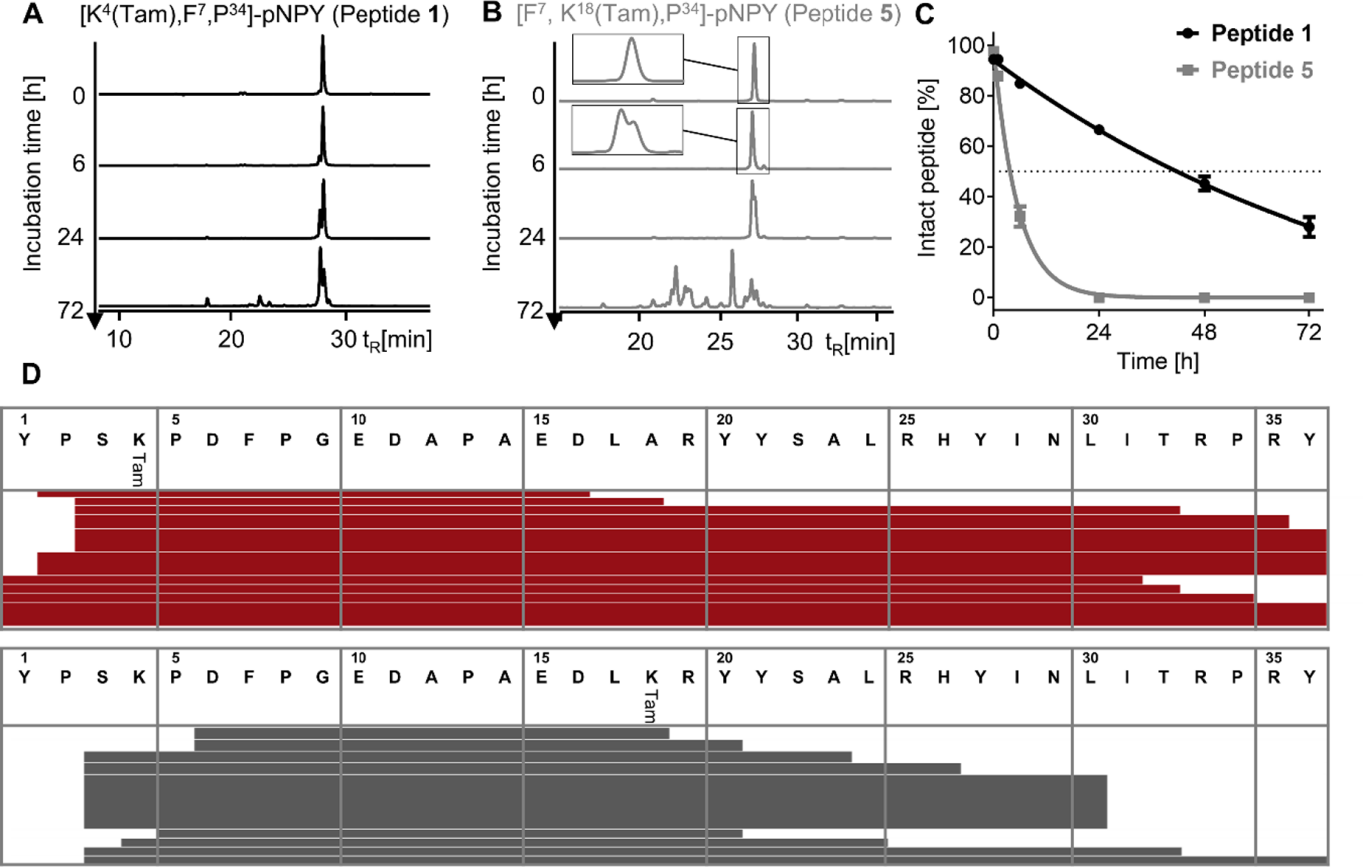
*S*tability assay of Tam-labeled NPY analogue peptides was performed in human blood plasma/DPBS (1:1). Peptide solutions (10 μM) were incubated at 37 °C and 500 rpm. Degradation of (A) [K^4^(Tam),F^7^,P^34^]-pNPY and (B) [F^7^,K^18^(Tam),P^34^]-pNPY was analyzed by using RP-HPLC at indicated time points by fluorescence detection and referenced to the control at 0 h (set at 100%). (C) Values represent the mean ± SEM of *n* = 4 independent experiments. (D) Comparison of detected cleavage positions using Lys^4^ or Lys^18^ in the [F^7^,P^34^]-pNPY sequence for Tam labeling. Horizontal range of the lines represent detected degradation products, and their vertical thickness lines refer to the relative quantity of the degradation product assessed by the area under the curve of the fluorescent channel in RP-HPLC.

Introducing fluorescence labels is a common method to analyze peptide stability because the proteolytic degradation can be easily quantified by RP-HPLC using fluorescence intensity tracking.^6,32^ To investigate their possible influence on peptide stability, we introduced Tam at position Lys^4^ (peptide **1**) or Lys^18^ (peptide **5**) to [F^7^,P^34^]-pNPY and compared the blood plasma stability (Figure 4). The graphic illustrates both the degradation products detected in mass spectrometry (displayed by the horizontal range of the lines) and their relative amount in the HPLC chromatograms (vertical thickness of the line). Peptide **5** exhibited a significantly reduced half-life time of 3.8 h (95% CI = 3.4–4.2 h) compared to peptide **1** with 43.5 h (95% CI = 39.2–48.5 h). In RP-HPLC, both chromatograms differ, and a comparison of the degradation patterns showed that peptide **5** had a stronger *N-*terminal truncation and cleavage sites were detected not only after Pro^2^ but also after Ser,^3^ Lys^4^, and Pro^5^ (Figure 4D). These positions were completely masked in peptide **1**. Also, the *C-*terminal truncation was stronger for peptide **5**, even though its Tam label was closer to the *C*-terminus than in peptide **1**.

These findings clearly show that attachment of Tam to Lys^4^ has a stabilizing effect on [F^7^,P^34^]-pNPY. Especially for DPP-4, cleaving *N*-terminal proline-consisting dipeptides, the accessibility seems to be reduced drastically if Tam is attached to Lys^4^. However, peptide accessibility is also reduced in the *C*-terminal region of peptide **1** compared to **5**, even far away from the label. The reason for this might be the PP-fold of the peptide, which already was published for NPY.^34,35^ Thus, hydrophobic Tam at position 4 could stabilize the PP-fold structure, which potentially masks cleavage sites in the middle of the peptide sequence. However, further investigation would be necessary to support this hypothesis, especially as the PP-fold in NPY is discussed controversially.^36^ Nevertheless, we could show with our data that introducing fluorescence labels at different positions in the sequence is crucial to recognizing possible influences on stability. However, as masking of cleavage sites can never be completely ruled out, alternative possibilities might be used to validate peptide stability.

### Investigation of Stability with Isotopically Labeled Peptides

**Figure 5:**
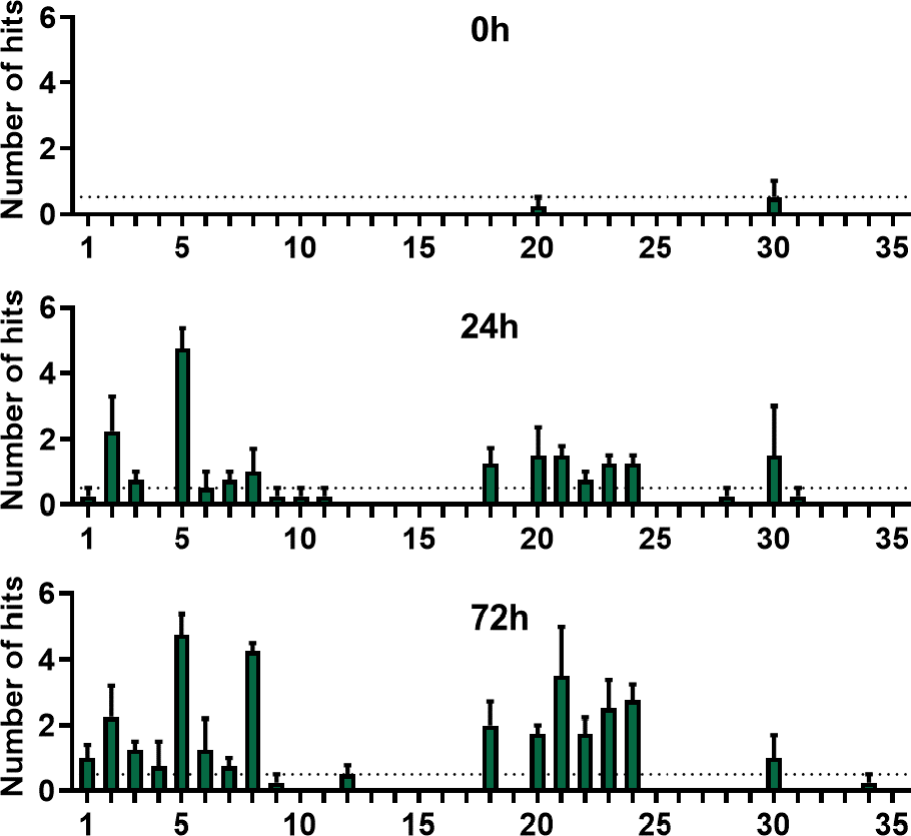
In vitro stability test of isotope-labeled and nonlabeled [F^7^,P^34^]-pNPY (1:2) was performed in human blood plasma/DPBS (1:1). Peptide solutions (10 μM) were incubated at 37 °C and 500 rpm. Degradation products were measured with nano-LC-ESI-MS and, following analysis, with the software *Proteome Discoverer 2.0*. Occurrence of cleavage sites was detected after 0, 24, and 72 h of incubation. *n* = 4.

An alternative way to assess peptide stability without affecting the chemical structure is by incorporating isotopically labeled amino acids and analyzing degradation by MS. Here, we mixed [F^7^,P^34^]-pNPY (peptide **6**) and isotopically labeled [F^7^,G^9^(^13^C_2_,^15^N),A^18^(^13^C_3_),L^30^(^13^C_6_,^15^N),P^34^]-pNPY (peptide **7**) in a ratio of 2:1 (*n/n*) and examined the blood plasma stability. Following nano-LC/MS/MS analysis, the data were processed with *Proteome Discoverer 2.0 Software*. Utilizing the peptide mixture provides an internal control to distinguish between peptide signals and background, as only hits found for both peptides were considered. In the 0 h sample, no degradation was measured, whereas after 24 h of incubation in blood plasma, no intact peptide was detected anymore, and the cleavage after Pro^5^ occurred most prominently (Figure 5). Furthermore, truncated peptides after Pro^2^ were measured. Interestingly, cleavages between Ala^18^ and Leu^24^ were also detected. After 72 h, the two cleavage areas in the *N-*terminal region and in the central peptide sequence occurred with increased abundance, and cleavage after Pro^8^ was observed additionally. Except for the cleavage after position 18, none of these cleavage positions were detected in peptide **1** (Figure 4). Yet, all cleavage positions were found in peptide **5** (Figure 4). The major cleavages after Pro^2^, Pro^5^, and Pro^8^ suggest DPP-4 activity after stepwise *N*-terminal shortening.^30,31^ The detected cleavage sites for the Lys^18^-labeled peptide **5** are more consistent with the cleavage sites from the isotopic approach than peptide **1**. We assume that cleavage in the central region of the peptide occurs only after *N-*terminal shortening and the associated structural change. This assumption is supported by the increased detection of cleavage sites between Lys^18^ and Leu^24^. The comparison of fluorescence and isotope labeling clearly elucidates the differences between the analysis and labeling methods of stability tests.

## Conclusions

Peptide stability is a significant research topic in various contexts. Predicting peptide degradation and identifying relevant degradation sites within the lead sequence are crucial for developing peptide therapeutics. Validating the peptide stability for assay setups with long incubation times is critical to prevent data misinterpretation. The literature offers a vast variety of diverse protocols for peptide stability assessments. However, according to our study, there is no one-size-fits-all methodology.

While MS can provide a good qualitative assessment of peptide degradation and detection of cleavage products, the application of LC-MS/MS is growing in popularity for quantifying peptides in biological fluids.^37,38^ However, this method is cost-intensive,^39^ and analysis of peptide stability with isotope-labeled peptides does not necessarily replace fast and cost-effective stability studies with fluorescent labels. Thus, a combined approach of both methods yields the most reliable results. In an early stage, identification of relevant degradation sites or control of long-term assays with fluorescent labels provides an important starting point for prediction of peptide stability. For the final qualitative analysis, LC-MS/MS with isotope-labeled amino acids provides data that are more reliable.

## Supporting Information Available

Peptide synthesis and characterization, nano-LC/MS/MS, and comparison of cleavage products (PDF)
