# Supplementary material for: Comparison of Protocols to Test Peptide Stability in Blood Plasma and Cell Culture Supernatants
Source: ACS Pharmacol Transl Sci. 2024 Oct 14;7(11):3618–25. doi: 10.1021/acsptsci.4c00503 (PMC11555501; doi:10.1021/acsptsci.4c00503)
Supplement: Supplementary file 1 — pt4c00503_si_001.pdf [file pt4c00503_si_001.pdf]

Supporting Information:

Title: Comparison of protocols to test peptide stability in blood plasma and cell culture supernatants

Authors: Anna Kohler[1]‡, Eva-Maria Jülke[1]‡, Jan Stichel[1], Annette G. Beck-Sickinger[1]*

* Corresponding author.

‡ These authors contributed equally.

Author affiliations: [1]Institute of Biochemistry, Faculty of Life Sciences, Leipzig University, Brüderstr. 34, 04103 Leipzig, Germany.

E-Mail-address of the corresponding author: abeck-sickinger@uni-leipzig.de

Method S1: Methods for Peptide synthesis

For *C*-terminal amidated peptides synthesis was carried out on NovaSyn TGR R resin (Merck), while peptides with free *C*-termini were synthesized on pre-loaded Wang resin (Iris Biotech). Synthesis was carried out in a combination of automated solid-phase peptide synthesis (SPPS) using the Syro II peptide synthesizer (MultiSynTech) and manual coupling. All reactions were carried out at room temperature and under shaking. For automated synthesis, coupling reactions were performed twice with 8 equiv. of the fluorenylmethoxycarbonyl (Fmoc)-protected amino acid (Iris Biotech) activated with equimolar amounts of oxyma (Iris Biotech) and *N,N'*-diisopropyl carbodiimide (DIC, Iris Biotech) in dimethylformamide (DMF, Biosolve) for 30 min. Automated Fmoc-deprotection was achieved by incubation with 40% (*v/v*) piperidine (Sigma-Aldrich) in DMF for 3 min and, subsequently, 20% (*v/v*) piperidine in DMF for 10 min. Manual coupling of amino acids and fatty acids was carried out with 3-5 equiv. Fmoc-protected amino acid, equivalent amount of 1-hydroxy benzotriazole (HOBt, Merck) and DIC in DMF for 3-16 h. Further, Fmoc was deprotected manually by incubating resins twice for 10 min in 20% (*v/v*) piperidine in DMF. For 1-(4,4-dimethyl-2,6-dioxocyclohex-1-ylidene)ethyl (Dde) deprotection, resins were incubated 12-times with 2% (*v/v*) hydrazine (Sigma-Aldrich) in DMF for 10 min. Deprotection of methoxytrityl (Mmt) was performed by 15 incubations in 2% trifluoroacetic acid (TFA, *v/v*, Merck), 5% triisopropylsilane (TIS, *v/v,* Sigma-Aldrich) in dichloromethane (DCM, Biosolve) for 1 min each. Tam was attached to the peptide by double coupling of 2 equiv. 6-carboxytetramethylrhodamine (Tam, emp biotech), 1.9 equiv. *O*-(7-azabenzotriazol-1-yl)-*N,N,N,N*-tetramethyluronium hexafluorophosphate (HATU, Sigma-Aldrich), and 2 equiv. *N,N*-diisopropylethylamine (DIPEA, Sigma-Aldrich) in DMF for 3-16 h. Isotope-labels were introduced in to the peptide sequence by manual coupling two-times 2 equiv. of the isotope-labeled amino-acid (eurisotop) with 2 eqiv. HOBt and 2 equiv. DIC in DMF overnight. Peptides were cleaved from the resin with 90% TFA (*v/v)*, 7% thioanisole (TA, *v/v*, Sigma-Aldrich), 3% thiokresole (TK, *v/v*, Sigma-Adlrich) or 90% TFA, 7% TA, 3% 2,2′-(ethylendioxy)-diethanthiol (DODT, *v/v*, Sigma-Aldrich) for 3 h. Subsequently, crude peptide was precipitated from ice-cold diethyl ether (Merck) for at least 30 min at -20°C. Further, the

crude was washed in cold diethyl ether three-times and dried in vacuum. Peptides were dissolved in 20-30% (*v/v*) acetonitrile (ACN, VWR) in H_2O. Purification was achieved by preparative reversed-phase high performance liquid chromatography (RP-HPLC, Hitachi) on an Aeris Peptide 5µ XB-C18-column (250 x 21,2 mm, 100 Å, 5 µm, flow-rate 15 ml/min, Phenomenex). A linear gradient of eluent B (0.1% (*v/v*) TFA in H_2O) in eluent A (0.08% (*v/v*) TFA in ACN) increasing by 1%/min over 20 min was used. Purity of peptides was validated by RP-HPLC on an Aeris Peptide 3,6u XB-C18-column (250 x 4,6 mm, 100 Å, 3,6 µm, flow-rate 1.55 ml/min, Phenomenex), and Jupiter 4u Proteo 90 Å C-12-column (250 x 4,0 mm, 90 Å, 4 µm, flow-rate 1 ml/min, Phenomenex). A linear gradient of eluent B in eluent increasing by 1.25%/min over 40 min at 40°C was utilized. Detection was performed at λ=220 nm and λ=280 nm. Relative areas under the curves were calculated from the chromatograms using integration by EZ Chrome Elite software (VWR). Further, matrix-assisted laser desorption/ionization time-of-flight (MALDI-ToF; Ultraflex III MALDI-ToF/ToF, Bruker Daltonics) as well as ESI-Orbitrap (Thermo Fisher Scientific) mass spectrometry was performed. For MALDI-ToF, peptides were cocrystalized with Super-DHB (Bruker) or α-cyano-4-hydroxycinnamic acid (Bruker) matrix as dried droplet. For ESI-Orbitrap mass spectrometry, peptide samples were diluted 1:20 in spray solution consisting of H_2O / 75% ACN / 0.1% formic acid (FA, *v/v/v*, Sigma -Aldrich). Spectra were obtained by direct infusion of the diluted peptides at 4 µl/min. Data acquisition was performed using the following parameters: resolution 120000 at m/z = 400, mass range 150-2000 m/z, acquisition time 1-3 min. Deconvolution of the obtained raw spectra was performed using the QualBrowser Software (Thermo Fisher Scientific).

Table S1: Analytical characterization of synthesized peptides. Peptide identity was verified by comparison of calculated monoisotopic mass (M_{mono}) und detected mass (M_{obs}) by (a) electrospray ionization-high capacity ion trap, (b) matrix assisted laser desorption ionization-time of flight or (c) electrospray ionization-orbitrap mass spectrometry. Compound purity and retention time (t_R) was analyzed by reversed phase-high performance liquid chromatography using an linear gradients of 20-70% (*v*/*v*) eluent B in eluent A over 40 min on (d) Aeris Peptide XB-C18 (250 × 4.6 mm, 3.6 µm, 100 Å, flow rate = 1.55 ml/min), (e) Kinetex Biphenyl (250 × 4.6 mm; 5 µm; 100 Å, flow rate = 1.55 ml/min) or (f) Jupiter Proteo (250 × 4.6 mm; 4 µm; 90 Å, flow rate = 0.6 ml/min). Abbreviations: Bip = biphenylalanine; Dpr = 2,3-diaminopropionic acid; Lau = lauric acid; Tam = 6-carboxytetramethylrhodamine. Oct = octanoic acid. Peptide 1 and 3 were previously described.[17,18]

	**Peptide**	**Sequence**	**Mass spectrometry**		**t_R [min]**		**Purity [%]**
			**M_{mono} [Da]**	**M_{obs} [Da]**	**Column 1**	**Column 2**	
**1**	[K^{4}(Tam),F^{7},P^{34}]-pNPY	YPSK(Tam)PDFPGEDAPAEDLARYYSALRHYINLITRPRY-NH$_2$	4665.3	$[M+4H]^{4+}$: 1167.6[a]<br>$[M+5H]^{5+}$: 934.3[a]<br>$[M+6H]^{6+}$: 778.8[a]<br>$[M+7H]^{7+}$: 667.7[a]<br>$[M+8H]^{8+}$: 584.4[a]	17.1[d]	20.0[e]	> 95
**2**	[K^{27}(Lau),K^{31}(Tam)]-sNPY$_{(27-36)}$	K(Lau)INPK(Tam)-Bip-RLRY-NH2	2003.2	$[M+1H]^{+}$: 2004.2[b]<br>$[M+2H]^{2+}$: 1002.6[b]	20.2[d]	24.0[f]	> 95
**3**	[Dpr^{3}, K^{16}(Tam)]-Ghr	GS-Dpr(Oct)-FLSPEHQRVQQRK(Tam)ESKKPPAKLQPR	3780.0	$[M+4H]^{4+}$: 946.0[c]<br>$[M+5H]^{5+}$: 757.0[c]<br>$[M+6H]^{6+}$: 631.0[c]<br>$[M+7H]^{7+}$: 541.0[c]<br>$[M+8H]^{8+}$: 473.5[c]	8.6[d]	11.8[f]	> 95
**4**	[Dpr^{3},K^{16}(Tam),K^{20}(Odd)]-Ghr	GS-Dpr(Oct)-FLSPEHQRVQQRK(Tam)ESKK(Odd)PPAKLQPR	4076.3	$[M+4H]^{4+}$: 1020.6[c]<br>$[M+5H]^{5+}$: 816.7[c]<br>$[M+6H]^{6+}$: 680.7[c]<br>$[M+7H]^{7+}$: 583.6[c]<br>$[M+8H]^{8+}$: 510.8[c]	16.8[d]	22.6[f]	> 95
**5**	[F^{7}, K^{18}(Tam),P^{34}]-pNPY	YPSKPDFPGEDAPAEDLK(Tam)RYYSALRHYINLITRPRY-NH$_2$	4722.3	$[M+4H]^{4+}$: 1182.1[c]<br>$[M+5H]^{5+}$: 945.9[c]<br>$[M+6H]^{6+}$: 788.4[c]<br>$[M+7H]^{7+}$: 675.9[c]<br>$[M+8H]^{8+}$: 591.4[c]	15.9[d]	17.9[f]	> 95
**6**	[F^{7},P^{34}]-pNPY	YPSKPDFPGEDAPAEDLARYYSALRHYINLITRPRY-NH$_2$	4253.1	$[M+1H]^{+}$: 4254.2[b]<br>$[M+2H]^{2+}$: 2127.6[b]	15.1[d]	17.1[f]	> 95
**7**	[F^{7},G^{9}($^{13}C_2$,^{15}N),A^{18}($^{13}C_3$), L^{30}($^{13}C_6$,^{15}N),P^{34}]-pNPY	YPSKPDFPG($^{13}C_2$,^{15}N)EDAPAEDLA($^{13}C_3$)RYYSALRHYINL($^{13}C_6$,^{15}N)ITRPRY-NH$_2$	4266.2	$[M+3H]^{3+}$: 1423.7[c]<br>$[M+4H]^{4+}$: 1068.1[c]<br>$[M+5H]^{5+}$: 854.6[c]<br>$[M+6H]^{6+}$: 712.4[c]<br>$[M+7H]^{7+}$: 610.8[c]	15.1[d]	17.1[f]	> 95

Method S2: Methods for Nano-LC/MS/MS

Reconstituted peptide solutions were separated on a ES902 EASY-spray RP-HPLC column (C18, 250 mm x 75 µm, 100 Å, 2 µm, Thermo Fisher Scientific) using a linear gradient eluent B (0.1% FA in ACN) in eluent A (0.1% FA in H_2O) of 20% to 70% over 120 min at 50°C column temperature. Data were acquired using a data-dependent LC/MS/MS method with HCD and fixed collision energy of 30%. For each full scan (m/z 200 – 1700, resolution = 120000 @ m/z 400), ten fragment ion scans were obtained starting with the most intense signal of the full scan (isolation width = 2 m/z). Data analysis was performed using ProteomeDiscoverer 2.0 Software (Thermo Fisher Scientific). The following parameters have been applied: Enzyme: Unspecific Cleavage; Precursor mass tolerance: 10 ppm; Fragment mass tolerance: 0.02 Da; Dynamic modifications: Oxidation (Δm = +15.995 Da); Isotopic Label (Glycine/$^{13}C(2)$, $^{15}N(1)$, Δm = +3.004 Da); Isotopic Label (Alanine/$^{13}C(3)$, Δm = +3.010 Da); Isotopic Label (Leucine/$^{13}C(6)$, $^{15}N(1)$, Δm = +7.017 Da); Amidated Protein *C*-Terminus (Δm = -0.984).

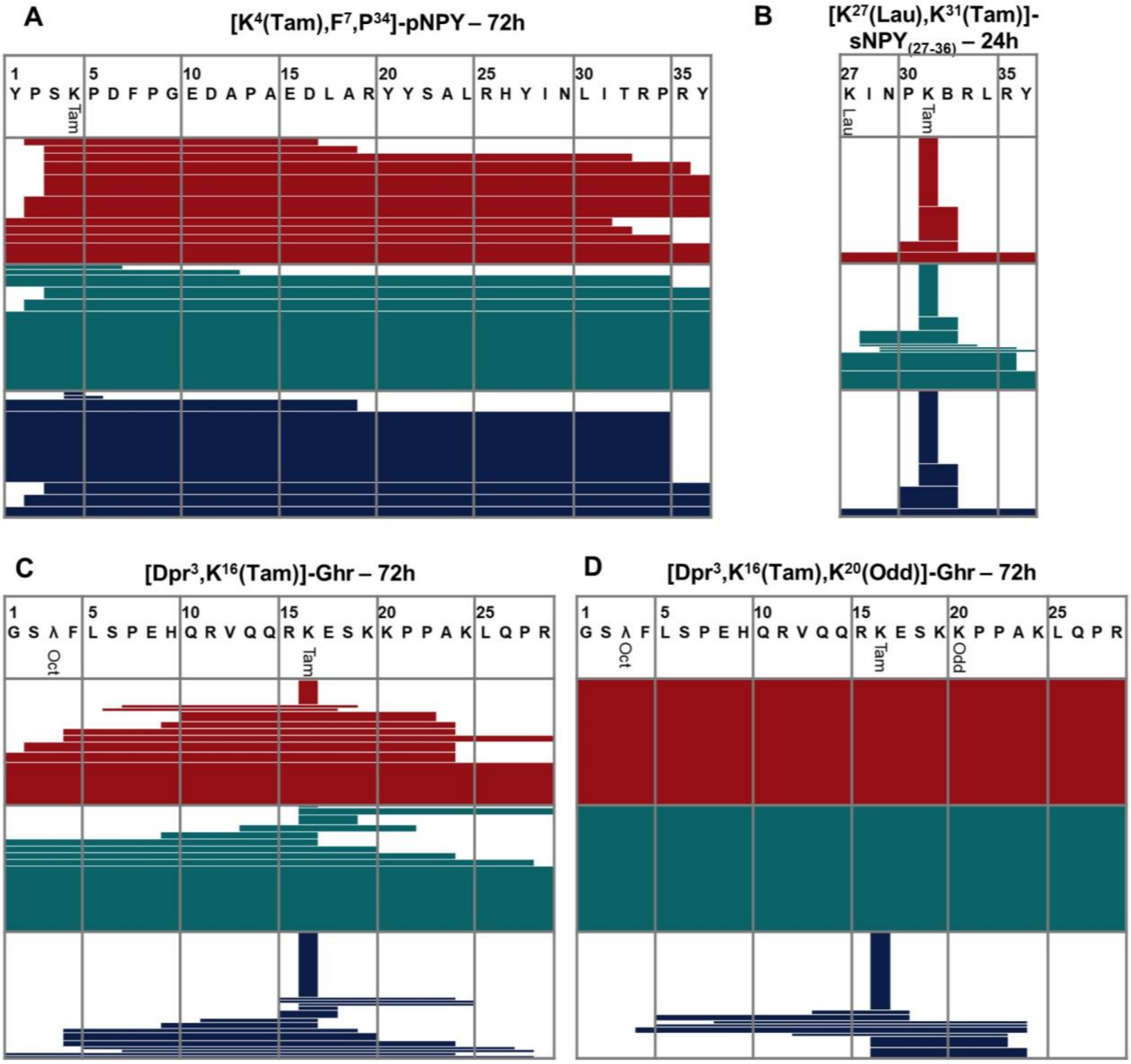

Figure S1: Comparison of cleavage products in different stability assays. The colored areas indicate the detected peptide fragments of **(A)** [K^{4}(Tam),F^{7},P^{34}]-pNPY, **(B)** [K^{27}(Lau),K^{31}(Tam)]-sNPY$_{(27-36)}$, **(C)** [Dpr^{3},K^{16}(Tam)]-Ghr and **(D)** [Dpr^{3},K^{16}(Tam),K^{20}(Odd)]-Ghr after incubation in **(red)** human blood plasma, **(blue)** HEK293 cell supernatant and **(green)** Calu-3 cell supernatant. The thickness of the lines refers to the relative quantity of the degradation product assessed by the area under the curve of the fluorescent channel in RP-HPLC.
